# Behavioral Advantages of the First-Person Perspective Model for Imitation

**DOI:** 10.3389/fpsyg.2016.00701

**Published:** 2016-05-17

**Authors:** Rui Watanabe, Takahiro Higuchi

**Affiliations:** ^1^Department of Cognitive Neurobiology, Graduate School of Medical and Dental Sciences, Tokyo Medical and Dental UniversityTokyo, Japan; ^2^The Japan Society for the Promotion of ScienceTokyo, Japan; ^3^Department of Health Promotion Science, Tokyo Metropolitan UniversityTokyo, Japan

**Keywords:** imitation, visual perspective, visuomotor information, stimulus–response compatibility, first person perspective

## Abstract

Visuomotor information may be better conveyed through a first-person perspective than through a third-person perspective. However, few reports have shown a clear behavioral advantage of the first-person perspective because of the confounding factor of spatial stimulus–response compatibility. Most imitation studies have utilized visuospatial imitation tasks in which participants use the same body part as that used by the model, identified by its spatial position (i.e., the response action is predefined). In such studies, visuomotor information conveyed by the model does not appear to facilitate imitative behavior. We hypothesized that the use of the first-person perspective would facilitate more efficient imitative behavior than a third-person perspective when participants are asked to choose and reproduce an action identical to that of the model rather than to select the same body part; this task requires the analysis of both visual and motor information from the model rather than a simple assessment of spatial information. To test this hypothesis, we asked 15 participants to observe a model from two perspectives (first-person and third-person) with left or right hand laterality and to lift their index finger with an identical movement type (extension or flexion) as quickly as possible. Response latencies were shorter and fewer errors were made in trials using the first-person perspective than in those using the third-person perspective, regardless of whether the model used the right or left hand. These findings suggest that visuomotor information from the first-person perspective, without confounding effects of spatial information, facilitates efficient imitative behavior.

## Introduction

During imitative behavior, the perspective from which an action is viewed affects the transfer of sensory information between the model and imitator (Sambrook, 1998; Meltzoff, 2005). Several imitation studies have reported that sensory information available from the first-person perspective (i.e., as if the imitator were observing the model from his/her own perspective) is greater than that viewed from the third-person perspective (i.e., with the model facing the observer; Vogt et al., 2003; Jackson et al., 2006; Oosterhof et al., 2012). The first-person perspective model facilitates more accurate imitative behavior than does the third-person perspective model (Jackson et al., 2006; Nishizawa et al., 2015; Ramenzoni et al., 2015) and induces greater activity in the mirror neuron system (MNS), which is implicated in the processing of visuomotor information (Watanabe et al., 2013). As shown by action observation studies, the first-person perspective visually transfers motor information from the models to observers (Alaerts et al., 2009; Wakita and Hiraishi, 2011), allowing observers to respond quickly and appropriately during a task (Bortoletto et al., 2013; Bach et al., 2014; Brattan et al., 2015). For instance, Brattan et al. (2015) asked participants to observe an action sequence video that was briefly interrupted by an occlusion image. A temporal shift was incorporated into the post-occlusion action sequence, and participants were asked to judge whether the time at which the action resumed was earlier or later than expected based on the occlusion period. Participants responded more accurately when observing the action from the first-person perspective than from the third-person perspective. The results of these previous reports imply that the first-person perspective induces strong visuomotor transformation between the model’s and the imitator’s actions (i.e., utilizes the direct matching system, in which a visual body image automatically activates a corresponding action representation; Vogeley and Fink, 2003; Vogt et al., 2003; Meltzoff, 2005; Jackson et al., 2006).

Although several recent studies have reported empirical behavioral data to support the advantage of the first-person perspective in imitative behavior, such effects are likely to be confounded by stimulus–response (S–R) compatibility. That is, responses are generally faster when the moving stimulus limb and the responding limb are compatible in some dimension (e.g., spatial location, anatomical characteristics, or movement direction) than when they are not (Prinz, 1997; Brass et al., 2000; Schmidt and Lee, 2005). For example, in the finger-tapping imitation task, the imitative response is generally faster when the spatial finger alignment (i.e., left or right) of the participant’s hand corresponds to that of the model (Brass et al., 2000; Bertenthal et al., 2006; Catmur and Heyes, 2011; Boyer et al., 2012; Mengotti et al., 2012). In addition, previous studies have reported that imitative behavior is affected by interactions among multiple dimensions of S–R compatibility between the model’s and participant’s limb (e.g., visual perspective and anatomical or spatial compatibility; Bertenthal et al., 2006; Catmur and Heyes, 2011; Boyer et al., 2012; Jimenez et al., 2012; Cooper et al., 2013); although the effects of spatial compatibility are confounded by anatomical incongruence between the model’s and imitator’s bodies, such interference is attenuated by the first-person perspective (Ramenzoni et al., 2015).

**FIGURE 1:**
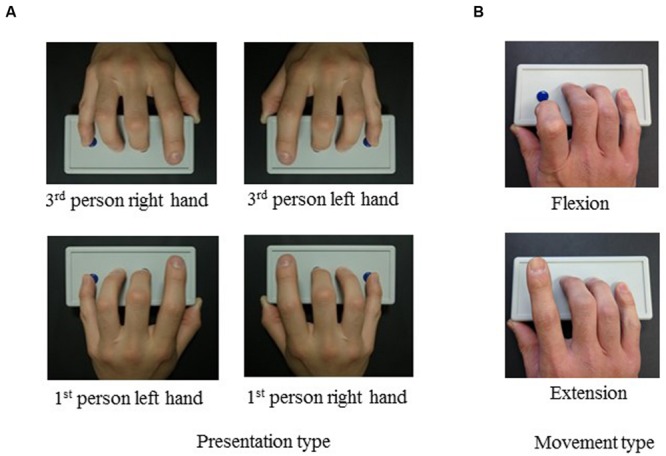
**(A)** Presentation types: visual perspective and hand laterality. **(B)** Movement types: when lifting with flexion or extension, the distal and proximal interphalangeal joints were flexed or extended, respectively.

Spatial compatibility is a confounding factor that is particularly difficult to address in imitative behavioral studies (Nishizawa et al., 2015; Ramenzoni et al., 2015). A study by Nishizawa et al. (2015) found that participants’ response limb movements and body direction were identical to those of a model when viewed from the first-person perspective, suggesting a behavioral advantage of this perspective. However, their results may have been confounded by spatial compatibility, as Boyer et al. (2012) have suggested that it is difficult to distinguish spatial and imitative effects in this standard stimulus–response imitative paradigm. Importantly, the results of our previous study (Watanabe et al., 2013), which failed to show behavioral advantages of the first-person perspective, may also have been confounded by spatial compatibility between the stimulus and participants’ hands. Participants using their right hands showed faster responses to first-person right hand and third-person left hand stimuli (i.e., those that were spatially congruent with participants’ responding right hand) than to the first-person left hand and third-person right hand (i.e., spatially incongruent stimuli; **Figure 1A**).

**FIGURE 2:**
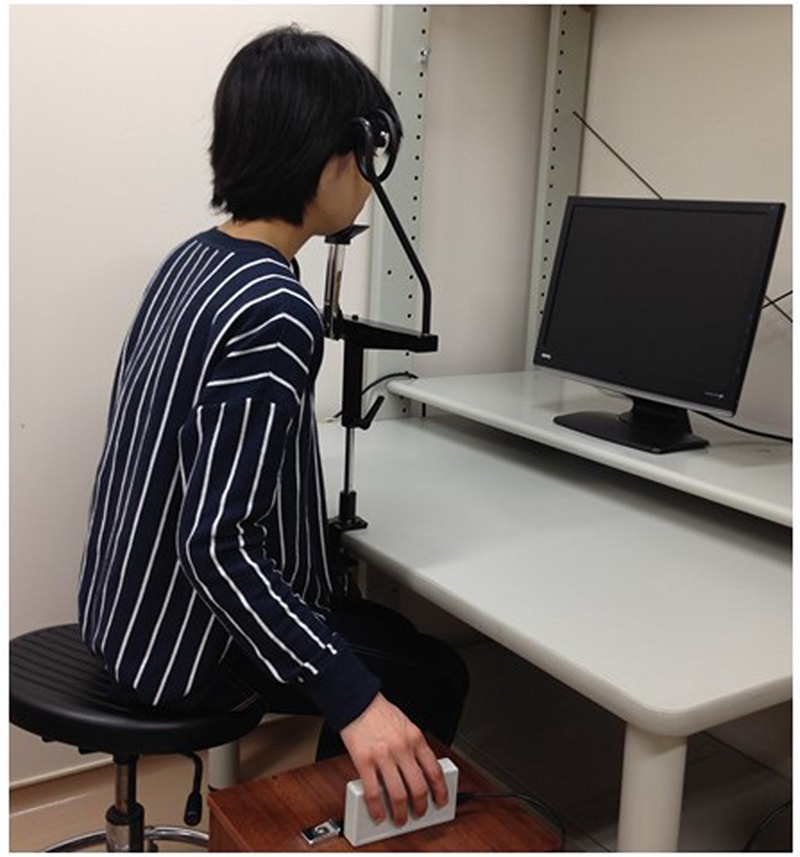
**Experimental design.** Stimulus and response right hands were placed in orthogonal planes to eliminate effects of spatial compatibility.

The purpose of the present study was to investigate the behavioral effects of the first-person perspective without the confounding effects of spatial compatibility. To accomplish this, we used an imitation task in which participants were asked to choose and reproduce an identical action to that of a model, and not just to choose the same body part as that used by the model. In addition, the participants’ response hands were placed orthogonally to that of the model in order to avoid direct effects of spatial compatibility between the model’s and participants’ postures (**Figure 2**). In previous studies, participants were asked to use an identical body part to that used by the model (e.g., index, middle, or ring finger); thus, the imitated movement was predetermined (e.g., finger-lifting or finger-tapping; Brass et al., 2000, 2001; Press et al., 2008; Boyer et al., 2012; Jimenez et al., 2012). Furthermore, the participants’ limbs were aligned in the same plane and orientation as that of the first-person model. Therefore, it is possible that participants needed to analyze mainly visuospatial information to select the identical body part with the aid of spatial compatibility, without transforming visuomotor information.

Additionally, participants in the present study were asked to imitate one of two movement types using their index fingers: extension or flexion of the distal and proximal interphalangeal joints (**Figure 1B**). There are minimal left–right visuospatial differences between these two movements. Additionally, the models involved two visual perspectives (first- and third-person) and right or left hand lateralities (**Figure 1A**). To elucidate the effect of visual perspective on behavior, we measured reaction times and movement errors in a choice reaction time (CRT) task. We also asked participants to perform a simple reaction time (SRT) task in which a predetermined finger movement was presented as a “go” signal; the SRT primarily represents the time required for execution of the finger movement.

Our previous imaging study demonstrated that activity in the MNS, which has been suggested to be related to visuomotor transformation (Grèzes et al., 2003; Rizzolatti and Craighero, 2004; Iacoboni and Dapretto, 2006), was maximized by the first-person perspective model, regardless of anatomical compatibility between the model’s and imitator’s hands (i.e., right or left hands; Watanabe et al., 2013). Based on these results, we propose that the primary effect of the first-person perspective is to convey visuomotor information from a model to facilitate imitation. Therefore, we hypothesized that, when one must choose and reproduce an identical action to that of a model, and not just to choose the same body part, the first-person perspective would facilitate more efficient imitative behavior (i.e., faster and more correct responses) than the third-person perspective. This result would reflect the use of visuomotor information conveyed through the first-person perspective to imitate the modeled behavior.

## Materials and Methods

### Participants and Ethics Statement

Fifteen healthy subjects (11 females and 4 males, mean age 27.6 ± 6.1 years) provided written informed consent to participate in the study, which was approved by the Institutional Ethics Committee of the Tokyo Medical and Dental University and the Tokyo Metropolitan University. The guidelines of the Declaration of Helsinki were also followed. None of the participants had any history of neurological or psychiatric illness. They were all right-handed, as assessed by the Edinburgh Handedness Inventory (Oldfield, 1971).

### Apparatus and Task

The models were presented using Presentation 14.0 (Neurobehavioral Systems, Inc., USA) and displayed on a 22-inch FlexScan monitor (1680 pixels × 1050 pixels, 24 bit color; EIZO, Inc., USA). Participants responded by pressing the leftmost button on a response box (4 Button Curve Right, Current Design, Inc., USA) with their index fingers.

**FIGURE 3:**
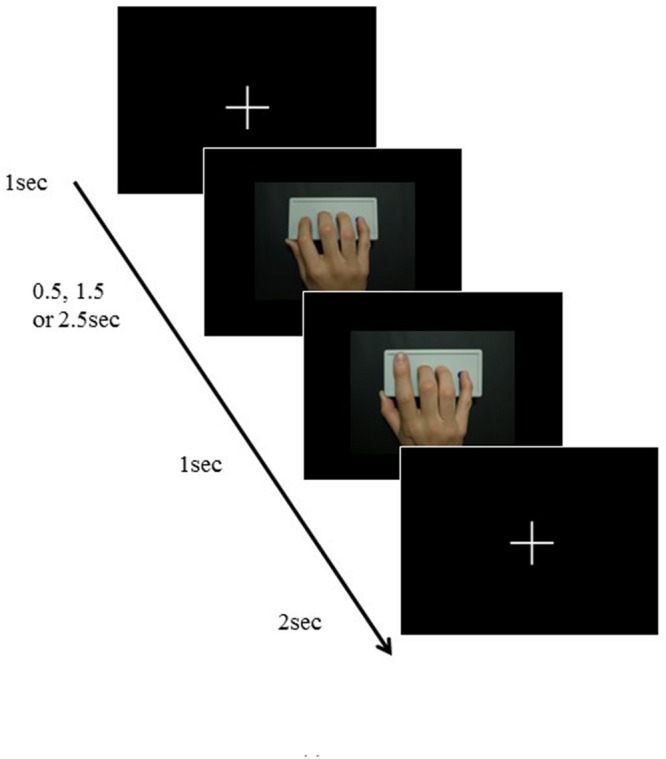
**Experiment time-course with examples of the model used in the experiment; extension movement shown from the first-person perspective**.

The modeled finger stimuli consisted of a set of video clips showing lifting of the index finger from a resting position on a response box with buttons. The visual perspective and the hand laterality of the model were manipulated so that the presented movement (a) was observed from either the first- or third-person perspective, and (b) used either the right hand (i.e., anatomically congruent with the participant’s right hand) or the left hand (i.e., anatomically incongruent; **Figure 1A**). Each presentation showed either extension or flexion of the distal and proximal interphalangeal joints (**Figure 1B**). Each video clip lasted for 4.5–6.5 s, consisting of presentation of a fixation point for 1 s, a static view of the pronated hand resting on the response buttons for 0.5, 1.5, or 2.5 s, finger movement for 1 s, and presentation of a fixation point for 2 s (**Figure 3**).

The participant’s goal was to imitate the modeled movement as quickly as possible by lifting the index finger of their right hand using the same movement as that shown by the model. Participants held the response box with the right hand, while the right arm was aligned with the right side of the trunk with no pronation or supination of the forearm, in order to reduce the effect of spatial S–R compatibility. This alignment placed the fingers of the model and the participants in orthogonal planes, as in our previous study (Watanabe et al., 2013).

### Procedure

Each participant sat on a chair facing a monitor placed 50 cm in front of him or her. Prior to performing the task, all participants performed a practice session to become familiar with the required actions. The practice session consisted of four blocks of ten trials each, with one of the four presentation types used consistently in each block. The order of the presentation types was randomized among the participants. None of the practice session trials were included in the statistical analysis.

The task was divided into two parts: a CR (choice reaction) task and an SR (simple reaction) task. Identical video clips were used in each task. In the CR task, the participant was asked to lift his or her index finger in an identical manner to that shown in the video, as quickly as possible. In contrast, in the SR task, the movement type of the finger was pre-stated; therefore, the participant’s task was simply to lift their index finger with the predetermined movement type as soon as the model’s finger was lifted in the video clip. The order of the two tasks was counterbalanced among the participants.

Each task included four blocks, each using one of the four presentation types. The order of the presentation types was randomized among the participants. Each block in the CR task comprised 84 trials, with 42 trials using each movement type. Each block in the SR task consisted of 42 trials and was divided into two sub-blocks of 21 trials each, showing extension or flexion movements. The type of finger motion was constant within each sub-block. The participants were informed before each sub-block of which movement type would be presented. A total of 504 trials (i.e., CR task: 84 trials × 4 blocks; SR task: 42 trials × 4 blocks) were presented for each participant. The participants’ responses were filmed with a digital video camera throughout the imitation task.

### Dependent Measures and Statistical Analyses

We measured CRTs and SRTs and calculated the error proportions. Errors in selecting the correct movement type were counted using the digital video recordings of the CR task. Response times shorter than 120 ms were classified as anticipation errors, and response times greater than three standard deviations above each participant’s average CRT were considered omission errors. These trials, as well as those in which the incorrect movement type was selected, were discarded from the RT analyses.

The main dependent measures were CRTs and error rates; the former reflects the time required for selection of the correct finger movement, which was based on visuomotor information from the model. The error rate represents the accuracy of the response selection based on the information shown in the model. Three-way repeated-measures ANOVA (visual perspective × hand laterality × movement) were used to compare the SRTs, CRTs, and error rates among the four presentation types.

## Results

**FIGURE 4:**
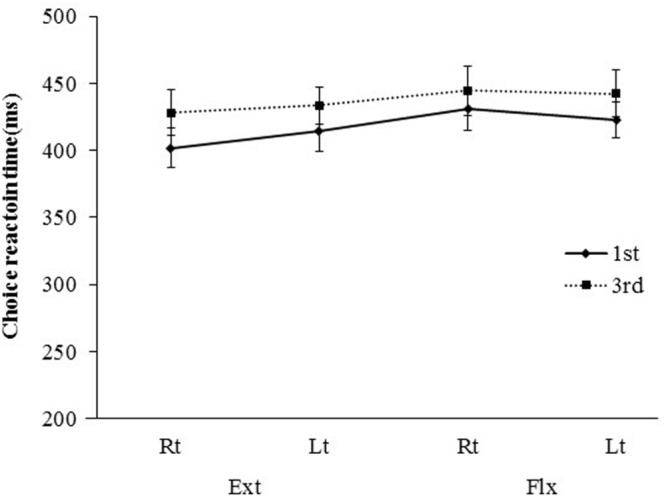
**Mean choice reaction time [CRT; ±standard error (SE)] for presentations differing in visual perspective, hand laterality, and movement type**.

**FIGURE 5:**
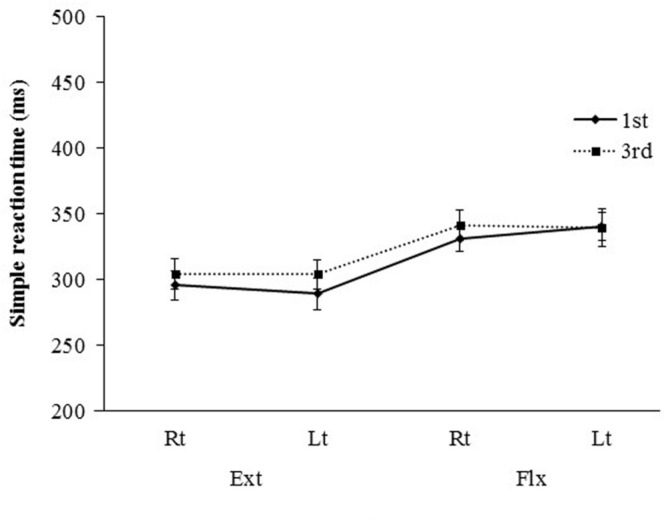
**Mean simple reaction time (SRT; ±SE) for presentations differing in visual perspective, hand laterality, and movement type**.

**FIGURE 6:**
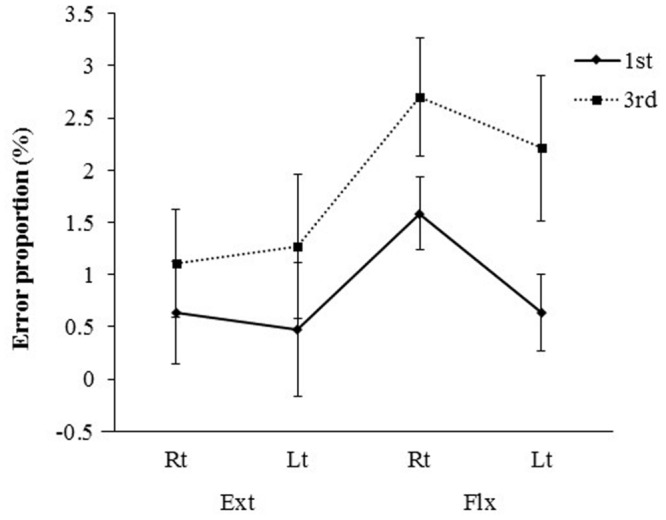
**Mean error rates (±SE) for presentations differing in visual perspective, hand laterality, and movement type**.

Mean (±standard error) CRTs, SRTs, and error rates for each combination of visual perspective, hand laterality, and movement type are shown in **Figures 4–6**, respectively. Three-way ANOVA of the CRT data revealed a significant main effect of visual perspective [*F*(1,14) = 7.96, *P* < 0.05, η^2^ = 0.13]. The mean CRT was shorter for the first-person perspective condition than for the third-person perspective condition (417 ms vs. 437 ms). There was also a significant interaction between hand laterality and movement type [*F*(1,14) = 5.81, *P* < 0.05, η^2^ = 0.02]. A follow-up test using the Ryan method indicated that, for right-hand presentations, the mean CRT for the extension condition was shorter than for the flexion condition (415 ms vs. 438 ms; *P* < 0.005). No other significant interactions were observed. There was also a significant main effect of movement type [*F*(1,14) = 7.52, *P* < 0.05, η^2^ = 0.09]. The CRT for the extension condition was shorter than for the flexion condition (419 ms vs. 435 ms). The main effect of hand laterality failed to reach significance [*F*(1,14) = 0.18, ns].

A three-way ANOVA revealed no significant differences in SRT according to visual perspective [*F*(1,14) = 1.66, ns] or hand laterality [*F*(1,14) = 0.008, ns]. However, a significant main effect of movement type was found [*F*(1,14) = 25.07, *P* < 0.0005, η^2^ = 0.41], with shorter SRTs for the extension condition than for the flexion condition (298 ms vs. 338 ms).

A three-way ANOVA of the error rate data revealed a significant main effect of visual perspective [*F*(1,14) = 9.67, *P* < 0.01, η^2^ = 0.066]. Error rates were significantly smaller for the first-person perspective condition than for the third-person perspective condition (0.83% vs. 1.82%). A significant main effect of movement condition was also found [*F*(1,14) = 4.82, *P* < 0.05, η^2^ = 0.055], indicating fewer errors in the extension condition than in the flexion condition (0.87% vs. 1.78%). The main effect of laterality failed to reach significance [*F*(1,14) = 1.24, ns], and none of the interactions were significant. The pattern of errors across conditions was similar to that of the CRTs, thus confirming that the results were not attributable to a speed-accuracy trade-off. Few errors were made in the SRT task regardless of presentation and movement types. The error rates in the SRT task were very low, with anticipation or incorrect movement errors occurring in less than 0.05% of all trials.

## Discussion

The present study investigated the effect of the first-person perspective on imitative behavior in the absence of spatial compatibility as a confounding factor. Consistent with our hypothesis, the behavioral results clearly demonstrated that the first-person perspective led to faster responses when participants were asked to reproduce the action of the model, regardless of whether the model’s right or left hand was shown. Moreover, participants made fewer errors when imitating actions viewed from the first-person perspective. In contrast, the response latency in the SR task, in which there was no need to discriminate between movement types, was not significantly affected by the first-person perspective. Overall, our data indicate that visuomotor information available from the first-person perspective models facilitates efficient imitative behavior when spatial compatibility is eliminated, regardless of anatomical compatibility between the model and imitator.

The robust effect of the first-person perspective on reaction times and errors in the CR task indicates that visuomotor information activated action representation in the participants. Action representations are a component of forward modeling for internal feedback, which enables us to predict a sensory outcome without actual performance of the action and is important to the control of response timing (Wolpert et al., 1995; Sakai et al., 2002; Choudhury et al., 2007). Action representations are coded in the frontal and parietal areas, which are involved in the processing of visuomotor information (Blakemore and Sirigu, 2003; Buccino et al., 2004; Nelissen et al., 2005). The processing of motor imagery obtained through the first-person perspective is particularly associated with action representation (Jeannerod, 1995). Thus, our results suggest that the first-person perspective provides robust visuomotor information that activates action representations, which in turn facilitate faster and more precise imitative behavior than does the third-person perspective. This effect of action representation was similar regardless of whether the model’s right hand or left hand was shown because imitative performances were not significantly different between the first-person right hand and left hand conditions. This result could reflect the fact that we always see our own moving left and right hands from a first-person perspective and receive sensory feedback accordingly (i.e., visuomotor learning), whereas we rarely experience our own movements from a third-person perspective. Such experience facilitates visuomotor transformation and the development of action representations for both the right and left hands. Accordingly, our findings suggest that the core role of the first-person perspective is to induce visuomotor transformation and activate action representations in order to produce efficient imitative behavior regardless of anatomical compatibility. The reduction of spatial compatibility effects in our behavioral paradigm supports the validity of this conclusion.

Our previous brain imaging study demonstrated the advantage of the first-person perspective in terms of brain activity (Watanabe et al., 2013), consistent with our interpretation of the first-person perspective effects observed in the current study. Presentation of an action from the first-person perspective induced strong brain activity in the MNS and the frontal-parietal network (specifically, the dorsal premotor area and superior parietal lobule), which are associated with visuomotor transformation and action representation (Grèzes et al., 1999; Rizzolatti and Matelli, 2003; Felician et al., 2004; Rizzolatti and Craighero, 2004; Blakemore et al., 2005; Brass and Heyes, 2005; Nelissen et al., 2005; Parkinson et al., 2010). Additional indirect evidence supports our interpretation of the visuomotor effects of the first-person perspective. Chaminade et al. (2005) showed that, when participants were asked to reproduce a movement using the same limb as a model presented from a first-person perspective (i.e., an action representation task), front-parietal and visual areas were activated, both of which are associated with visuomotor transformation.

Considering the present findings, the effects of the first-person perspective reported by previous imitation studies were likely influenced by the degree of dynamicity of the model’s action. Nishizawa et al. (2015) asked participants to imitate whole-body movements that were likely highly dynamic and conveyed rich visuomotor information through the first-person perspective; this dynamicity may have contributed to the observed positive effects of the first-person perspective despite the fact that they did not control for anatomical or spatial compatibility. Conversely, Ramenzoni et al. (2015) asked their participants to synchronously imitate continuous action sequences (simple tapping movements with the index finger), a task involving spatial mapping and temporal demands rather than visuomotor transformation. Thus, when an imitation model includes less-dynamic visuomotor information, the first-person effect seems to be attenuated. Additionally, their behavioral data may have been influenced by anatomical and spatial compatibility. In the SR task, in which the response movement was predefined, there was no behavioral advantage attributable to the first-person perspective as compared to the third-person perspective. Previous studies using simple imitative reaction tasks (i.e., automatic imitation tasks) have reported that predetermined responses are initiated faster when cued by compatible vs. incompatible actions (e.g., finger-tapping responses cued by finger tapping rather than by finger lifting; Brass et al., 2001) because a compatible model provides images conveying the sensory feedback of an action, which facilitate imitative behavior. Press et al. (2008) used an automatic imitation task in which the participants executed pre-instructed hand movements (opening or closing) in response to actions performed by a model. Their results demonstrated that response latency is not modulated by the modeled hand laterality when there is no need to identify the modeled movement type before imitation. Based on these results, our SR task included only the compatible condition; in this paradigm, the first-person perspective did not lead to faster responses despite the strong visuomotor transformation elicited by this perspective. This finding suggests that only when response selection is required (i.e., in the CR task) does robust visuomotor information available from a first-person perspective provide a greater behavioral advantage.

## Conclusion

The results of the present study confirm that visuomotor information available from a first-person perspective facilitates efficient imitation behavior. This conclusion is supported by our data showing that movements observed from the first-person perspective without confounding effects of spatial compatibility engendered faster responses and fewer errors in a movement selection task, regardless of the laterality of the model hand. The observed effects add to previously acquired data illustrating the facilitative effects of the first-person perspective on imitative behavior.

## Author Contributions

Study conception and design: RW and TH. Acquisition of data: RW. Analysis and interpretation of data: RW. Drafting of manuscript: RW and TH.

## Conflict of Interest Statement

The authors declare that the research was conducted in the absence of any commercial or financial relationships that could be construed as a potential conflict of interest.
